# Usability of a novel cholangiopancreatoscope for tumor detection and stone management

**DOI:** 10.1016/j.vgie.2025.12.008

**Published:** 2025-12-26

**Authors:** Shuji Mitsuhashi, Chinmay Guralwar, Rishad Khan, Samuel Han, Jad P. AbiMansour, Eric J. Vargas, Bret T. Petersen, Vinay Chandrasekhara

**Affiliations:** Division of Gastroenterology and Hepatology, Mayo Clinic, Rochester, Minnesota, USA

## Abstract

**Background and Aims:**

Peroral cholangiopancreatoscopy (POCP) is increasingly used in complex ERCP but remains limited by small working channels and maneuverability challenges. This case series describes early user experience with a novel single-use POCP system for evaluation of indeterminate biliary strictures and electrohydraulic lithotripsy (EHL) of pancreatic and biliary stones.

**Methods:**

A case series included individuals undergoing evaluation for indeterminate biliary strictures and complex stone management. Procedures were evaluated for technical success, usability (Likert scale), diagnostic yield, and adverse events (AEs).

**Results:**

Twelve patients were included. Among the 9 who underwent cholangioscopy-directed biopsy samples, all provided adequate tissue. Of 6 malignancies, none were diagnosed by brush cytology, whereas cholangioscopy-directed biopsy samples were diagnostic in 4 of 5 cases of cholangiocarcinoma and 1 case of hepatocellular carcinoma. In all 3 stone cases, a 4.5F (catheter/probe size unit; 1F = 0.33 mm) EHL probe was successfully advanced, achieving complete stone clearance in a single session. Five endoscopists performed the procedures, reporting high Likert scores for pushability, tip control, device passage, and image quality. No periprocedural AEs were noted.

**Conclusions:**

In this limited single-center, noncomparative experience, the system enabled tissue sampling and EHL with reported ease of use. These preliminary findings should be interpreted cautiously and in the context of standard ERCP risks, including pancreatitis, infection, perforation, and cholangitis.

## Introduction

**Figure 1:**
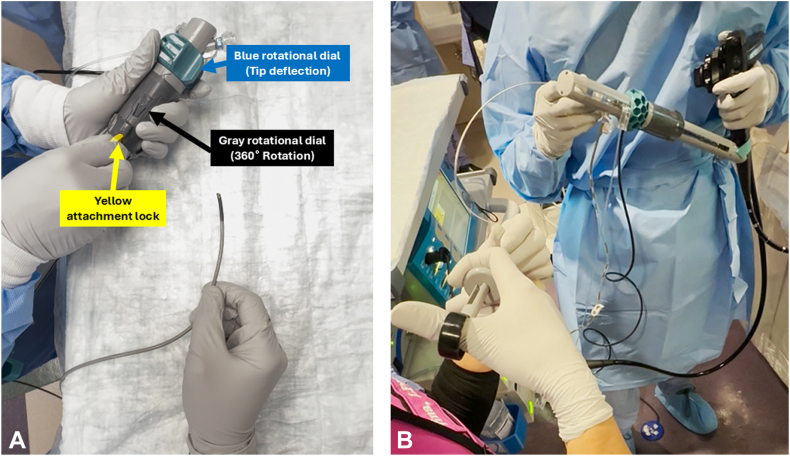
Novel peroral cholangiopancreatoscopy system components and procedural set-up. **A,** Close-up of the handle demonstrating the yellow attachment lock, gray rotational dial for 360° scope rotation, and blue dial for tip deflection. **B,** In-room procedural set-up showing scope handling and coordination.

Peroral cholangiopancreatoscopy (POCP) has revolutionized the evaluation and management of biliary and pancreatic diseases, yet conventional platforms are limited by small working channels and suboptimal maneuverability.^1^, ^2^, ^3^ The Dragonfly POCP system (Dragonfly Endoscopy Inc, Englewood, Colo, USA) (Fig. 1) is a novel single-use platform featuring enhanced tip control and a 1.7-mm working channel, which is substantially wider than current devices. This system accommodates larger accessories such as rotating forceps with a 6-mm open jaw span and 4.5F electrohydraulic lithotripsy (EHL) probes, expanding its potential utility in both diagnostic and therapeutic settings (Table 1).^4^^,^^5^ We describe our early experience using this system in 2 contexts: (1) cholangioscopy-directed biopsy of indeterminate biliary strictures and (2) EHL of complex pancreatic and biliary duct stones (Video 1, available online at www.videogie.org).

**Table 1:** Comparison of technical specifications between evaluated novel cholangioscopy system and standard digital single-operator system

Category	Feature	Evaluated novel system	Standard digital single-operator system
Scope characteristics	Working channel diameter	1.7 mm	1.2 mm
	Tip deflection	Up/down ±210°; lateral flexion available	Up/down ∼30°
	Scope rotation	360° continuous rotation	None
	Outer diameter	10.5F (∼3.5 mm)	10.5F (∼3.5 mm)
	Irrigation capacity	Higher (because of larger channel cross-section)	Moderate
Biopsy forceps compatibility	Jaw span (open width)	6.0 mm	4.1-4.5 mm
	Cup volume	Large-volume cups	Moderate-volume cups
	Cup geometry	Deep, serrated cups	Shallow to moderate depth
	Rotational capability	Yes	No
	Sheath diameter	1.6-1.7 mm	1.1-1.2 mm
Lithotripsy probe compatibility	Maximum compatible probe size	4.5F EHL probe	1.9-2.6F EHL probe

*EHL*, Electrohydraulic lithotripsy.

## Case presentations

### Case series 1: biliary strictures

**Figure 2:**
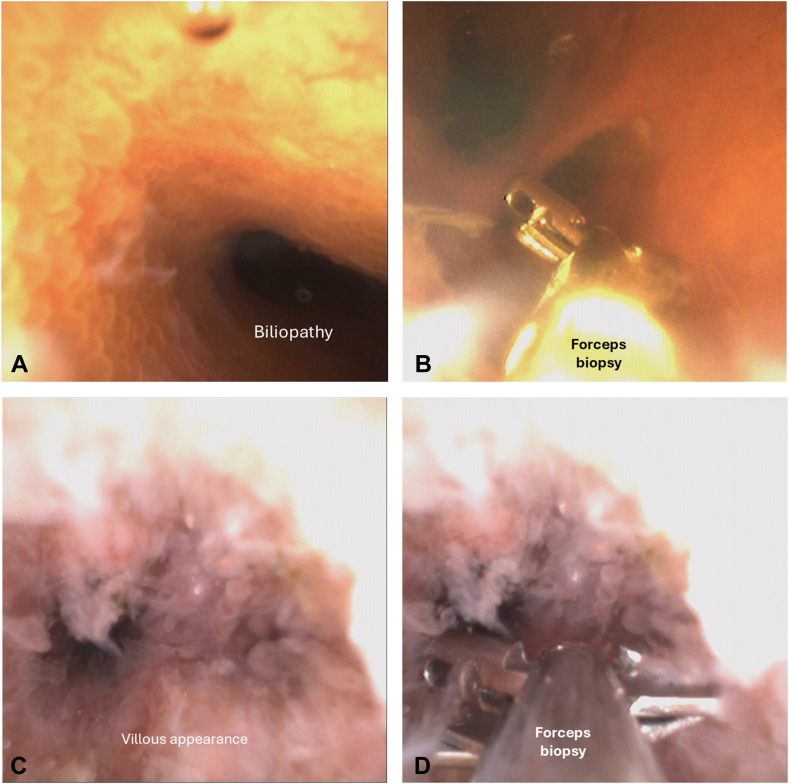
Novel cholangioscopy-directed forceps biopsy for biliary stricture. **A,** Endoscopic view of a benign biliary stricture caused by biliopathy. **B,** targeted biopsy using rotating forceps in the same benign case. **C,** endoscopic view of a malignant biliary stricture with villous appearance. **D,** targeted biopsy performed in malignant biliary stricture.

Nine patients (aged 24-77 years) with indeterminate biliary strictures underwent cholangioscopy using the novel POCP system, as summarized in Table 2. Concerning cholangioscopic findings including mucosal irregularity, villous projections, and neovascularization were observed in 6 cases. Targeted biopsy specimens were obtained using large-caliber rotating forceps (Fig. 2), and all procedures were technically successful. Biopsy specimens were histologically adequate in all cases, with a mean tissue area of 0.17 ± 0.21 cm^2^.

**Table 2:** Biliary stricture case summary: correlation of cholangioscopy, cytology, biopsy, and adverse events

Case	Malignant-appearing imaging with cholangioscopy	Brush cytology	Specimen size (cm^2^)∗	Biopsy result	Final diagnosis	Periprocedural adverse events
#1	Yes	Suspicious	0.06	Positive	Cholangiocarcinoma	No
#2	No	Atypical	0.24	Atypical	Portal biliopathy	No
#3	Yes	Suspicious	0.12	Positive	Cholangiocarcinoma	No
#4	Yes	Atypical	0.1	Positive	Hepatocellular carcinoma with biliary invasion	No
#5	Yes	Suspicious	0.08	Positive	Cholangiocarcinoma	No
#6	No	Negative	0.72	Negative	Indeterminate biliary stricture	No
#7	Yes	Atypical	0.07	Atypical	Cholangiocarcinoma	No
#8	Yes	Atypical	0.12	Positive	Cholangiocarcinoma	No
#9	No	Atypical	0.04	Negative	High-grade intraductal papillary neoplasm of the bile duct	No

Malignant-appearing imaging with cholangioscopy revealed findings including mucosal irregularity, villous projections, and neovascularization.

∗ Specimen size reflects the largest fragment.

Final diagnoses included 5 cases of cholangiocarcinoma (CCA), 1 hepatocellular carcinoma (HCC) involving the bile duct, 1 portal biliopathy, and 1 high-grade intraductal papillary neoplasm of the bile duct. One patient remained with an indeterminate biliary stricture, with both cytology and biopsy results negative. Among the 6 malignant cases (5 CCA and 1 HCC), brush cytology was either suspicious or atypical but not diagnostic. In 1 case, both brush cytology and biopsy specimen were atypical and nondiagnostic; however, the patient was ultimately diagnosed with CCA based on clinical follow-up, including malignant-appearing cholangioscopic findings, fluorescence in situ hybridization polysomy, elevated carbohydrate antigen 19-9, and imaging consistent with CCA. All 3 patients with nonmalignant final diagnoses had negative or atypical cytology and biopsy results, consistent with benign disease.

Endoscopists evaluated the system using a 5-point Likert scale (5 = best). Median ratings were 5 for pushability, 4 for tip control, 5 for device passage, and 5 for image quality. No periprocedural adverse events (AEs) were reported.

### Case series 2: pancreatic and biliary duct stones

**Figure 3:**
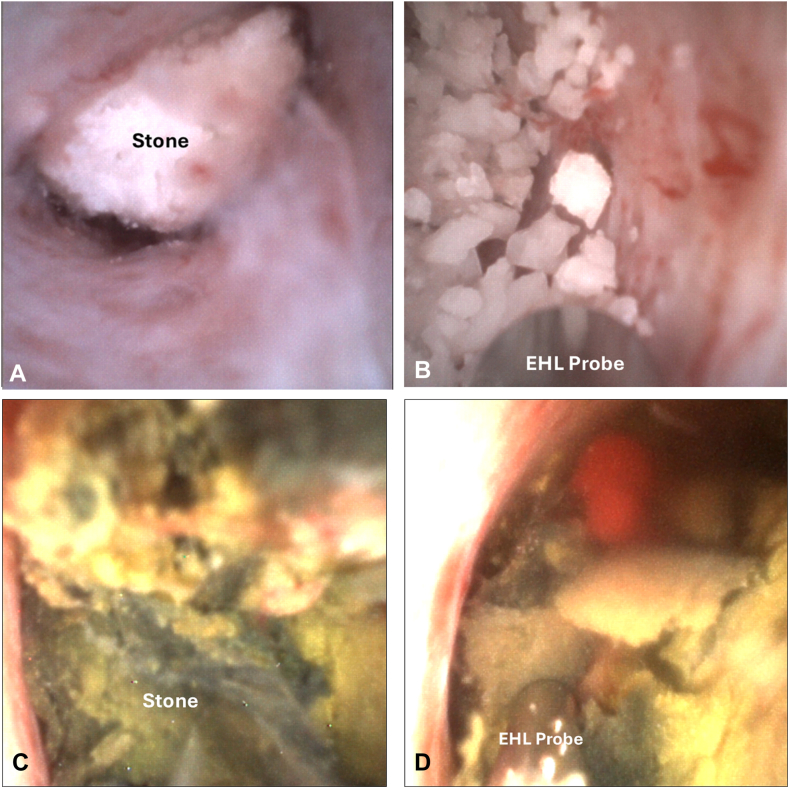
Novel cholangiopancreatoscopy-guided electrohydraulic lithotripsy in pancreatic and biliary ducts. **A,** Direct visualization of pancreatic duct stone. **B,** postlithotripsy appearance of pancreatic duct stone fragments. **C,** direct visualization of biliary stones. **D,** postlithotripsy appearance of stone fragments.

Three patients underwent POCP-guided EHL with the novel device, as summarized in Table 3. Two had obstructive pancreatic duct stones in the setting of chronic calcific pancreatitis, and 1 had choledocholithiasis with a malignant biliary stricture. In all cases, the 4.5F (catheter/probe size unit; 1F = 0.33 mm) EHL probe was successfully advanced through the working channel and duct without difficulty (Fig. 3). Lithotripsy was completed in a single session, followed by stone clearance using balloon or basket extraction and stent placement. Average procedure time was 99 minutes (range, 79-128). Endoscopists rated the system highly for EHL compatibility, maneuverability, and visualization (Likert score = 5 for all domains). There were no periprocedural AEs.

**Table 3:** Stone case summary: procedure details, outcomes, and adverse events

Case	Stone location	Time of the procedure	Successful stone clearance	Periprocedural adverse events
#10	Body/tail of pancreatic duct	128 min	Yes	No
#11	Body/tail of pancreatic duct	89 min	Yes	No
#12	Common bile duct	79 min	Yes	No

## Discussion

This case series highlights the novel POCP system's versatility and practicality in both diagnostic and therapeutic applications. For indeterminate biliary strictures, it enabled high-quality tissue acquisition with superior yield than conventional cytology. For challenging pancreatic and biliary stones, the larger working channel facilitated effective lithotripsy using robust probes, supporting single-session clearance. Consistently high Likert scores across multiple operators reflected favorable handling, maneuverability, accessory compatibility, and image quality, which likely contributed to the procedural success and absence of AEs observed in this limited experience. These early observations suggest that this novel POCP system may serve as a useful platform for complex pancreatobiliary interventions. Larger studies are warranted to further evaluate performance and long-term outcomes.

## Patient consent

All patients provided written informed consent for publication of their case details under approved Institutional Review Board #25 to 000491.

## Disclosure

The following authors disclosed financial relationships: S. Han is a consultant for Boston Scientific; E. J. Vargas is a consultant for Medtronic and receives research funding from Philips; J. P. AbiMansour has intellectual property with Ruhof Inc; B. T. Petersen is a consultant for Olympus America and Pentax, Inc, and a grant recipient and study participant for Boston Scientific and Ambu, Inc; V. Chandrasekhara is a consultant for Boston Scientific, receives research support from Merck, TaeWoong Medical USA, and Dragonfly Endoscopy, and is a stockholder in Nevakar Corporation; all other authors disclosed no financial relationships. The authors have no personal financial relationship with the company manufacturing this cholangiopancreatoscope, Dragonfly Endoscopy. All equipment, including the scope, biopsy forceps, and lithotripsy probes, was provided by the manufacturer under a research agreement for this study.

## Supplementary material

Video 1 Demonstration of device setup, procedural steps, and detailed postoperative course.
